# Predictors of not maintaining regular medical follow-up after stroke

**DOI:** 10.1186/s12883-023-03262-y

**Published:** 2023-06-20

**Authors:** Mellanie V. Springer, Lesli E. Skolarus, Chunyang Feng, James F. Burke

**Affiliations:** Stroke Program, University of Michigan Medical School, 1500 E. Medical Center Drive, Ann Arbor, Michigan, MI 48109-5855 USA

## Abstract

**Background:**

Regular medical follow-up after stroke is important to reduce the risk of post-stroke complications and hospital readmission. Little is known about the factors associated with stroke survivors not maintaining regular medical follow-up. We sought to quantify the prevalence and predictors of stroke survivors not maintaining regular medical follow-up over time.

**Methods:**

We conducted a retrospective cohort study of stroke survivors in the National Health and Aging Trends Study (2011–2018), a national longitudinal sample of United States Medicare beneficiaries. Our primary outcome was not maintaining regular medical follow-up. We performed a cox regression to estimate predictors of not maintaining regular medical follow-up.

**Results:**

There were 1330 stroke survivors included, 150 of whom (11.3%) did not maintain regular medical follow-up. Stroke survivor characteristics associated with not maintaining regular medical follow-up included not having restrictions in social activities (HR 0.64, 95% CI 0.41, 1.01 for having restrictions in social activities compared to not having restrictions in social activities), greater limitations in self-care activities (HR 1.13, 95% CI 1.03, 1.23), and probable dementia (HR 2.23, 95% CI 1.42, 3.49 compared to no dementia).

**Conclusions:**

The majority of stroke survivors maintain regular medical follow-up over time. Strategies to retain stroke survivors in regular medical follow-up should be directed towards stroke survivors who do not have restrictions in social activity participation, those with greater limitations in self-care activities, and those with probable dementia.

Stroke affects 795,000 Americans per year, and approximately 20% of strokes are recurrent [1]. Control of vascular risk factors, such as hypertension, is key to reducing recurrent stroke risk. The stroke survivor’s primary care physician (PCP) or a specialist physician largely carries the responsibility of managing vascular risk factors in the immediate post-stroke period and over time. Having a PCP may reduce the risk of stroke recurrence [2]. Hospital 30-day readmission rates are lower for stroke survivors who have a primary care visit within that time frame [3, 4]. A post-discharge PCP visit may allow for the detection and outpatient management of post-stroke complications [5].

Despite the known importance of regular medical follow-up visits, little is known about the prevalence and predictors of whether a stroke survivor does not maintain regular medical follow-up after stroke. According to national US data, ~ 60% of stroke patients have a primary care visit within 30 days of hospital discharge [3, 4]. Demographic and clinical predictors of having a PCP visit within 30 days include older age, female sex, discharge with home health care, and diabetes [3]. However, little is known about reasons stroke survivors establish but do not maintain regular medical follow-up over time. It is possible that stroke patients with vascular cognitive impairment, caused by underlying cerebral small vessel disease or the acute stroke lesion itself, might be less likely to maintain regular medical follow-up after stroke [6]. We used a longitudinal US dataset, the National Health and Aging Trends study, to determine the prevalence and predictors of whether a stroke survivor does not maintain regular medical care.

## Methods

### Study population

We identified all adult stroke survivors in the National Health and Aging Trends Study (NHATS) from 2011 to 2018. The NHATS is an annual interview of a nationally representative longitudinal sample of community dwelling Medicare beneficiaries (age 65 years or older) that collects information about demographics and ability to perform different activities. Participants are followed longitudinally over time and an additional cohort of participants was added in 2015. Stroke survivors were defined as those who answered ‘yes’ to the question “Has a doctor told you that you had a stroke?”. The NHATS interview in which stroke was self-reported can be considered the baseline interview. We only included post-stroke NHATS interview data. We excluded stroke survivors without NHATS visits after baseline because it would not have been possible to measure our primary outcome variable, whether a stroke survivor maintained regular medical follow-up. NHATS participants with proxy responses or without longitudinal data were excluded due to missing important variables. We also excluded any stroke survivors who never had visits with a regular doctor after stroke (answered no to the question ‘Is there a doctor that you think of as a regular doctor?’) throughout the observation period.

### Outcome

Our primary outcome was not maintaining regular medical care in the outpatient setting. Stroke survivors who initially answered ‘yes’ to the question “Is there a doctor that you think of as a regular doctor?” and answered ‘no’ to the question in a later interview year were classified as not maintaining regular medical care. Participants were censored at death or at the time of their last interview.

### Exposures

Demographic variables extracted from the NHATS baseline interview included education (less than high school, high school, and at least some post-high school), sex (male/female), and race/ethnicity (White, Black, Hispanic, or Other. Other race included American Indian/Asian/Native Hawaiian, Pacific Islander or Other specified race). Age and marital status were extracted from the NHATS interview during which the participant was identified as having not maintained regular medical care. Age was categorized as 65–69, 70–74, 75–79, 80–84, 85–89, and 90 years old or older. Married (yes/no) was defined as married or living with a partner vs. all other marital status categories (separated/divorced/widowed/never married).

Social determinants of health variables were taken from the NHATS interview during which the participant was identified as having not maintained regular medical care. Transportation to medical services (yes/no) was defined as ‘yes’ if the participant responded yes to either of the following questions “Does the place where you live offer residents a van or shuttle to doctors or other medical care providers?” or “Since the time of the last interview, did you drive yourself places?”. Financial hardship (yes/no) was defined as being present if the response was yes to the question “There are several state and federal programs [food stamps, other food assistance such as Meals-on-Wheels, or gas, electricity, or other energy assistance] that help people in need. In the last year, did you receive help from any of these programs?”. Care companion (yes/no) was defined as a yes response to the question “In the last year, did anyone sit in with you and your doctor during your visits?”. Restrictions in participation in social activities (yes/no) was defined as a yes response to any of the following questions “In the last month, did your health or functioning keep you from… visiting with friends and family not living with you? attending religious services? participating in clubs, classes, or other organized activities? going out for enjoyment?” and the participant identified the activity as being ‘very or somewhat important’.

Clinical variables were taken from the NHATS interview during which the participant was identified as having not maintained regular medical care. Dementia was categorized as probable dementia, which was a self-reported diagnosis of dementia or ≤ 1.5 standard deviations (SD) below the mean on 2 of orientation, memory, or executive function cognitive domains evaluated with a cognitive test battery or a score of 2 or higher on the AD8 dementia screening interview, an interview administered to an informant to screen for dementia; [7] possible dementia, which was defined as scoring ≤ 1.5 SDs below the mean on 1 of orientation, memory, or executive function cognitive domains; and no dementia which was defined as not meeting criteria for probable or possible dementia. The sum of chronic conditions was the total (0 to 8) of self-reported diagnoses of heart attack, heart disease, high blood pressure, arthritis, osteoporosis, diabetes, lung disease, or cancer. Limitations in household activities (equivalent to instrumental activities of daily living (IADLs)) were summed from 0 to 4 for each instrumental activity that the participant required help to perform over the previous month including laundry, grocery shopping, meal preparation, and handling bills/ banking. Limitations in self-care activities (equivalent to activities of daily living (ADLs)) were summed from 0 to 7 for each activity of daily living that the participant required help to perform over the previous month including eating, bathing, dressing, toileting or that the participant required help to perform over the past year including getting out of bed, getting around the home, or leaving home. Depression (yes/no) was defined as being present if the participant scored 3 or higher on the Patient Health Questionnaire-2, which is a 2-question screening test for depression. Anxiety (yes/no) was defined as being present if the participant scored 3 or higher on the Generalized Anxiety Disorder-2, which is a 2-question screening test for anxiety. This study was approved by the University of Michigan Institutional Review Board. Informed consent was not required for this study as the data are deidentified and publicly available.

### Statistical analysis

We performed descriptive statistics for the entire cohort of stroke survivors included in the analysis. Frequencies (percentage) were calculated for categorical variables. Means and standard deviations were calculated for continuous variables.

We estimated a cox regression model to predict the time to failing to maintain regular medical follow-up from demographic, social determinants of health, and clinical exposure variables. Missing data were handled using last observation carried forward simple imputation for missing quantitative variables. Missing values of categorical variables were replaced by the median for ordinal variables, and by the mode or last observation carried forward for nominal variables. Approximately 10% of data were missing.

Posthoc pairwise correlations were performed between exposure variables to identify correlated exposure variables to aid in interpretation of the final regression model. While some variables were correlated, as anticipated, the magnitude of the correlation was sufficiently modest that multicollinearity was unlikely to bias the results.

Data were analysed using SAS, version 9.4.

## Results

### Population description

**Fig. 1:**
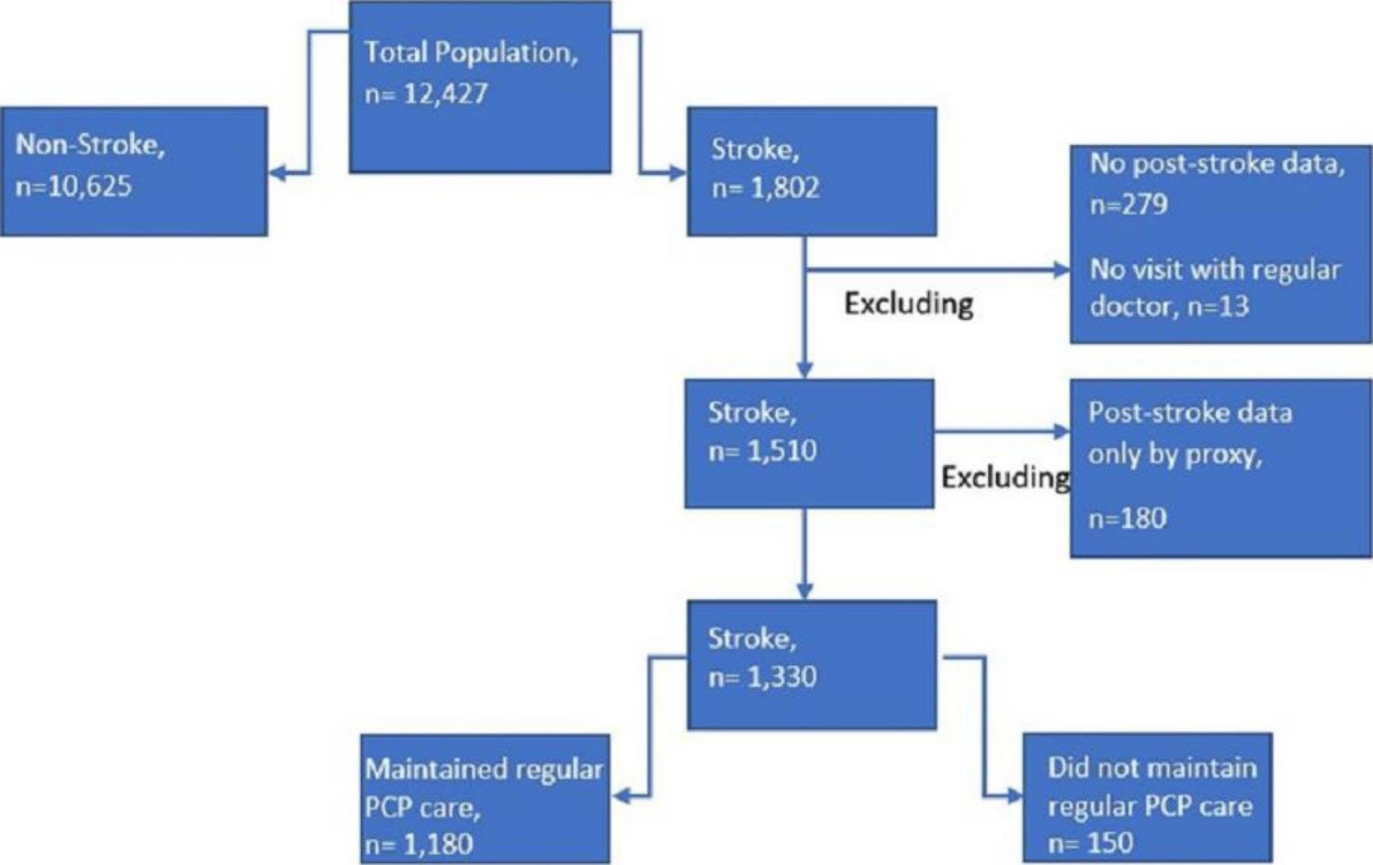
Flow chart of included and excluded participants

There were 1,802 participants who self-reported a history of stroke during the study period. Of these participants, 13 were excluded because they did not report having a regular doctor during the study period, 279 were excluded because they did not have NHATS interview data after their baseline interview and 180 participants were excluded because they only had interview data by proxy. Therefore, there were a total of 1330 participants included in the analysis (Fig. 1).

The greatest proportion of stroke survivors were in the age categories 80–84 and 75–79 years old (23% and 21% of stroke survivors respectively). Of the stroke survivors, 58% were female, 67% were White, 24% were Black, and 6% were Hispanic. Less than half of stroke survivors were married (44% married). The majority of stroke survivors had transportation to medical services (61%) and a companion accompanying them to doctor’s visits (63%). A small proportion of stroke survivors reported financial hardship (19%). The majority of stroke survivors had no dementia (61%), probable dementia was present in 24% of stroke survivors, and possible dementia was present in 15% of stroke survivors. Stroke survivors had a mean (± standard deviation) of 3.2 (± 1.6) chronic conditions, 2.4 (± 2.4) out of 7 limitations in self-care activities, and 1.5 (± 1.6) out of 4 limitations in household activities. Approximately one quarter of stroke survivors screened positive for depression (26%) and 21% screened positive for anxiety (Table 1). Of the stroke survivors, 150 (11.3%) did not maintain regular PCP care.

**Table 1:** Characteristics of study population of adult stroke survivors at baseline in the National Health and Aging Trends Study (2011–2018) (N = 1330)

Age (years)	N (%)
65–69	148 (11.1)
70–74	237 (17.8)
75–79	275 (20.7)
80–84	306 (23.0)
85–89	207 (15.6)
90+	157 (11.8)
Sex	
Female	776 (58.4)
Race/ethnicity	
White	886 (66.6)
Black	320 (24.1)
Hispanic	84 (6.3)
Other	40 (3.0)
Marital status	
Married	584 (43.9)
Education	
Less than high school	410 (30.8)
High school	408 (30.7)
At least some post high school	512 (38.5)
Social Determinants of Health	
Transportation to medical services	817 (61.4)
Financial hardship	259 (19.5)
Care companion	842 (63.3)
Restriction in participation in social activities	441 (33.2)
Clinical	
Probable Dementia	317 (23.8)
Possible Dementia	200 (15.0)
No Cognitive Impairment	813 (61.1)
Depression	342 (25.7)
Anxiety	280 (21.1)
	Mean (± standard deviation)
Chronic conditions	3.2 (± 1.6)
Limitations in self-care activities^a^	2.4 (± 2.4)
Limitations in household activities^b^	1.5 (± 1.6)

^a^value ranged from 0 to 7 ^b^Value ranged from 0 to 4. Higher numbers represent more limitations.

### Predictors of not maintaining regular medical follow-up

Three factors were associated with not maintaining regular medical care (Table 2). Restrictions in social activity participation was associated with lower risk of not maintaining regular medical care (hazard ratio (HR), 0.64, 95% confidence interval (CI) 0.41, 1.01). More limitations in self-care activities was associated with higher risk of not maintaining regular medical care (HR 1.13, 95% CI 1.03, 1.23). Stroke survivors with probable dementia compared to those with no dementia had increased risk of not maintaining regular medical care (HR 2.23, 95% CI 1.42, 3.49).

**Table 2:** Predictors of stroke survivors not maintaining regular medical care in the National Health and Aging Trends Study (2011–2018)

Exposure	Hazard ratio (confidence interval)	p-value
Age (years) (Reference = 65–69)		
70–74	0.65 (0.31, 1.36)	0.26
75–79	0.54 (0.26, 1.13)	0.10
80–84	0.56 (0.28, 1.15)	0.12
85–89	0.83 (0.41, 1.69)	0.61
90+	0.76 (0.36, 1.63)	0.48
Sex (Reference = female)		
male	1.24 (0.87, 1.78)	0.24
Race or ethnicity (Reference = White)		
Black	0.72 (0.47, 1.10)	0.13
Hispanic	1.42 (0.66, 3.06)	0.37
Other	1.36 (0.69, 2.67)	0.38
Education (reference = less than high school)		
high school	0.78 (0.51,1.20)	0.26
Some post-high school	0.76 (0.50, 1.15)	0.19
Married (reference = no)	0.83 (0.56,1.22)	0.34
Transportation to medical services (reference = no)	0.83 (0.56, 1.23)	0.34
Financial hardship (reference = no)	0.74 (0.47, 1.15)	0.18
Care Companion (reference = no)	0 (0, incalculable)	0.97
Restriction in participation in social activities (reference = no)	0.64 (0.41,1.01)	0.05
Dementia (reference = no dementia)		
Probable Dementia	2.23 (1.42, 3.49)	0.001
Possible Dementia	1.45 (0.89, 2.34)	0.14
Number of chronic conditions (range 0–8)	0.97 (0.88, 1.08)	0.62
Household activity limitations (range 0–4)	1.16 (0.99, 1.36)	0.08
Self-care activity limitations (range 0–7)	1.13 (1.03, 1.23)	0.01
Depression (reference = no)	1.37 (0.91, 2.06)	0.14
Anxiety (reference = no)	0.70 (0.42, 1.17)	0.17

## Discussion

In this US national dataset, we found that 11.3% of stroke survivors with an established regular medical doctor did not maintain regular medical care. Predictors of not maintaining regular medical care included having fewer restrictions in participation in social activities, greater limitations in self-care activities, and probable dementia.

Despite that restrictions in social activity participation was positively correlated with limitations in self-care activities, they were associated with not maintaining regular medical care in opposite ways. Limitations in self-care activities suggests greater dependency on others. Restriction in participation in valued social activities is not tied to dependency. Rather, the presence of social support and mobility have been associated with participation in valued activities after stroke [8]. It is possible that stroke patients, who lack dependency on others for transportation, choose to attend valued social activities supported by friends and family over medical office visits. More research is needed to clarify the factors underlying the observed relationship between restriction in social activity participation and regular medical care.

Probable dementia increased stroke survivor risk of failing to maintain regular medical care. Caregiver burden due to the complexity of care associated with caring for a stroke survivor with dementia might be contributing to the stroke survivor’s inability to maintain regular medical care. Stroke survivors with probable dementia or physical impairment are more likely to attend medical visits with a companion [9]. Given that caring for stroke survivors with greater functional disability and memory problems is associated with caregiver burden, [10] it is possible that the caregiver has difficulty sustaining the demands of the caregiving role which might contribute to the inability of stroke survivors with probable dementia to maintain regular medical care. Increasing caregiver social and physical support is a strategy that may enhance the physical and psychosocial well-being of stroke survivor caregivers to meet the demands of the challenging caregiver role [11].

Strengths of this study include the use of a national US database and its longitudinal design. Limitations include that stroke was self-reported, although self-reported stroke has been validated against clinical assessment [12] and that the NHATS measure of having a regular doctor is based on self-report rather than objective medical visits. Since we excluded stroke survivors who never had visits with a regular doctor after stroke, it is possible that stroke survivors less likely to engage with the US health care system are underrepresented in our sample. However, stroke survivors who never had visits with a regular doctor were less than 1% of our sample. As the sample was selected from a US national database, more research is needed to determine whether our findings apply to stroke survivors in other countries. We did not have data on stroke subtype. An area of future research could be whether predictors of maintaining regular medical follow-up after stroke vary with stroke subtype.

## Conclusions

Primary care is recommended for all stroke patients [5]. While it is important to establish a follow-up plan with a regular medical doctor at hospital discharge, stroke patients’ hospital discharge protocols should equally address the importance of maintaining regular medical care.

## Data Availability

The datasets used in the current study are available at https://www.nhats.org/researcher/data-access.

## Abbreviations

ADL: activities of daily living
IADL: instrumental activities of daily living
NHATS: National Health and Aging Trends Study
PCP: primary care physician
